# Effects of Different Farrowing Environments on the Behavior of Sows and Piglets

**DOI:** 10.3390/ani10020320

**Published:** 2020-02-18

**Authors:** Xiaojun Zhang, Congcong Li, Yue Hao, Xianhong Gu

**Affiliations:** State Key Laboratory of Animal Nutrition, Institute of Animal Sciences, Chinese Academy of Agricultural Sciences, Beijing 100193, China; zhangxiaojun14@163.com (X.Z.); congcongli1988@sina.com (C.L.); haoyueemail@163.com (Y.H.)

**Keywords:** farrowing system, sloping wall, nest material, sow, piglet

## Abstract

**Simple Summary:**

The farrowing crate has long been a severe welfare problem of sows as its space restriction greatly impairs the behavioral expression of sows and induces long-term stress. We aimed to investigate the behavioral pattern of sows and piglets reared under different farrowing systems a farrowing crate and free farrowing pens with sloping walls with or without nest materials. Sows and piglets in free farrowing pens had a higher level of activity and lower level of performing abnormal behaviors. Providing nest material enhanced the beneficial effect of free farrowing system on the behavior of sows and piglets. These results encourage us to introduce the free farrowing systems to the pig industry and thus improve the welfare condition of farrowing sows. However, improved managements should be investigated as the piglet loss in the free farrowing systems tended to be higher than that in the farrowing crate system.

**Abstract:**

We investigated the effect of different farrowing systems on the behavior of sows and piglets. In total, 22 hybrid sows (Yorkshire × Landrace) were randomly assigned into three farrowing systems, farrowing crate (FC), free farrowing pen with sloping walls (FFS), and free farrowing pen with sloping walls and nest materials (FFSN). The FFS and FFSN sows were more active, and exhibited less abnormal behaviors compared with the FC sows before and after parturition. FFS and FFSN piglets were more active compared with FC piglets. The increased activity of the FFS and FFSN sows might encourage the activity of their piglets, resulting in more proximity to sow behavior of their piglets. Providing nest materials improved the investigative behavior of sows and piglets. The total mortality of piglets in the free farrowing systems tended to be higher compared with the farrowing crate system. In conclusion, free farrowing system has beneficial effects on the behavior of sows and piglets but extra care in management needs to be taken to avoid piglet loss. Adding nest material in the farrowing pens is suggested to enrich the behavioral pattern of sows and piglets.

## 1. Introduction

For the sake of easier daily sow and piglet managements, efficient space utilization, and lower piglet mortality, farrowing crates were developed and have been widely used around the world [1,2]. Farrowing crates are highly restrictive systems, stopping sows from performing natural behavior such as walking, nest-building, and explorative behavior [3,4,5]. Sows confined in farrowing crates have impaired welfare state evidenced by investigations on stress responses and abnormal behaviors [6,7]. This restrictive management of sows is highly criticized as a result of the growing animal welfare awareness of public consumers. Under increasing societal pressure, various alternative farrowing accommodations were created and studied aiming to abolish the conventional farrowing crates [2]. For example, farrowing systems of outdoor farrowing systems [8], non-confined indoor farrowing systems (such as hillside/sloped pens [9], mushroom pens [10], and simple/well designed pens [11]), and group systems [12,13] were continuously studied. Based on a large amount of credible data, the mortality of live-born piglets pre-weaning between loose farrowing systems and farrowing crates had no big difference [2] but with inevitable extra economic investment. By evaluating those different farrowing systems with the welfare design index (WDI), Baxter et al. [2] concluded that farrowing systems of well-designed pens appeared to offer the best indoor alternative to conventional farrowing crates. 

The design of farrowing pens to avoid piglet crushing from sows lying down were intensively investigated. Damm et al. [14] gave a detailed description of the relation between lying down and rolling behavior of sows and piglet crushing. Fast-rolling and unsupported lying down seem to be the most dangerous for piglets crushing [1,14]. Specifically, in terms of the lying behavior of sows, Marchant et al. [1] observed a high percentage (89%) of lying was performed leaning against walls whereas only 11% of lying was conducted in the open area. If given a chance, sows would often take advantage of a solid surface to lean against when lying down [15]. Lying down against a surface allows the sow to better locate the piglets and providing support features could often provide the piglets an escape area at the bottom [1,14]. There were several studies that applied this rail or sloping “furniture” in their alternative farrowing systems such as the Werribee Farrowing Pen with sloping panels on three sides of the nest area [11], farrowing pen with rail/sloping wall [1], and modified Schmid pen with rail positioned along the back wall of resting area [16]. Cronin et al. [11] reported no difference in the number of piglets born per sow, the number born alive, stillborn, fostered, weaned, or the number of pre-weaning deaths of live-born piglets between the Werribee Farrowing Pens and the conventional farrowing crates. Marchant et al. [1] mainly focused on the lying performance of sows in the farrowing systems. However, the detailed behavioral study of sows and piglets kept with or without the rail/slope wall in the farrowing system is scarce. Therefore, we created an open farrowing system with sloping panels on three sides of the wall and compared the behavior of sows and piglets reared in this system with those reared in the conventional farrowing crates.

In addition, nest material was reported to have a “cushion effect” in improving the comfort level of sows and thus reduce their rolling frequencies [14]. The provision of nest material provided an opportunity for recreation of pigs and increased nest-building behavior of sows [7,17]. Hence, besides the conventional farrowing crate and the open farrowing system with slope panels, a third treatment of adding nest material at the backside of the free farrowing pens with slope panels was also involved in the current study. The aim of this study was to examine the effects of different farrowing environments on the piglet production and loss as well as the behavior of sows and piglets.

## 2. Materials and Methods

The experimental Animal Care Committee of the Institute of Animal Sciences, China Academy of Agricultural Sciences has approved the conduction of the current experiment under the accession number of IAS20160615.

### 2.1. Animals and Treatments

The experiment was conducted in a piggery in Yanqing District, Beijing, China. A total of 24 hybrid sows (Yorkshire × Landrace) which had similar body condition and on their third parity were randomly allocated to three treatments: farrowing crate (FC, N = 8), free farrowing pen with sloping walls (FFS, N = 8) and free farrowing pen with sloping walls and nest materials (FFSN, N = 8). Before being introduced to the experimental farrowing systems, the sows were all reared loose housed and were kept in groups of 15 heads. During the experiment, one sow from FFS and FFSN treatment was sick and was excluded, leaving seven sows in FFS and FFSN groups each. Sows were introduced into the house with farrowing units from day 7 before estimated parturition to day 22 after actual parturition (0–1 d before/after the estimated parturition). Farrowing house temperature was maintained at 20–22 °C during the first week after parturition and 18–20 °C during the rest of the experimental periods. All sows were fed three times a day, at 07:00, 13:00 and 18:00, and received 3.5 kg of a lactation diet (14.7 MJ/kg, dry matter, 16.0% crude protein,) daily from day 7 to day 4 before parturition, 2.5 kg per day on days 3, 2, and 1 before parturition, 1 kg on the day of parturition and then 0.5 kg extra per day onwards to a maximum of 8 kg on the 14th day after parturition. On day 4 after parturition, male piglets were castrated under analgesia and isoflurane anesthesia. Both sows and piglets had free access to water.

**Figure 1:**
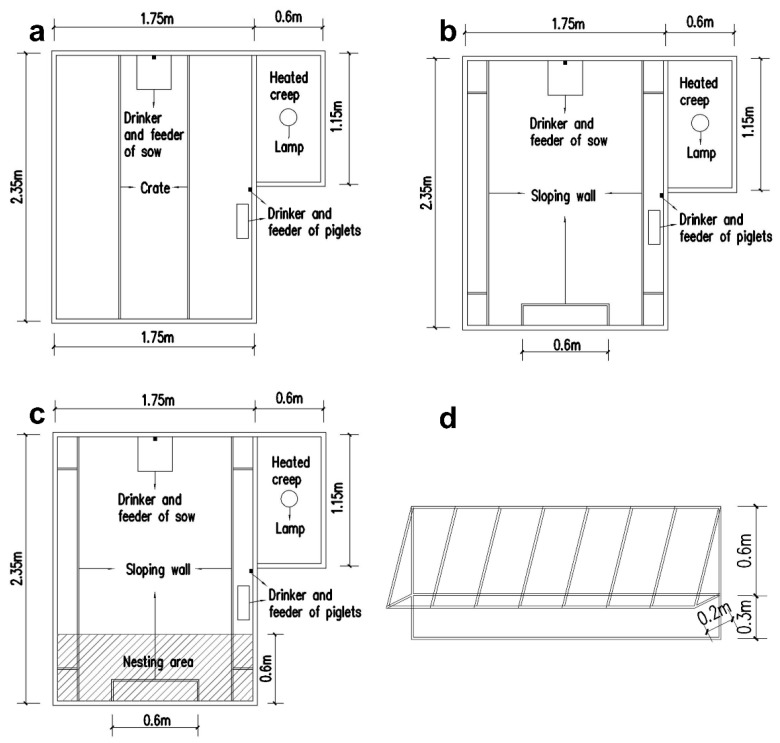
The diagram of the farrowing systems and the sloping wall. (**a**) Farrowing crate (FC); (**b**) Free farrowing pen with sloping walls (FFS); (**c**) Free farrowing pen with sloping walls and nest materials (FFSN); (**d**) Sloping wall.

We provided a diagram to show the construction of each farrowing system in Figure 1. All farrowing pens occupied an area of 2.35 m × 1.75 m, and a feeder was positioned in the middle of each rectangle-shaped farrowing pen. There was a piglet nursery box of 1.15 m × 0.6 m with a solid floor, and each nursery box was equipped with one heat lamp. For the FC system, a 0.6 m width of the crate was situated at the width middle of each pen (Figure 1a). For the FFS system, there was no crate in the pen, and sloping walls (made from rigid steel pipe) were fixed on three sides of the wall (Figure 1b) which serve as supporting fixtures for the lying of sows. In terms of the FFSN system, all the constructions were the same with the FFS system, except that there was a nesting area (1.75 m × 0.6 m) littered with the mix of rice husk and sawdust (the equal amount of each) at the backside of each pen with 0.6 m depth beneath pen floor level (Figure 1c). The bottom of the sloping walls was 30 cm above the floor and 20 cm away from the walls (Figure 1d). The pen floor of all farrowing systems was iron slotted floor except for the nesting area of the FFSN system.

### 2.2. Piglet Production and Loss

The number of piglet born, born alive, stillborn, weaned, being crushed were recorded throughout the experimental period. The total mortality per litter was calculated with the total death number divided by total born number and expressed as percentages. The crushing rate per litter, also express as percentages, equals to the crushing number divided by the number of piglet born alive. In addition, the birth weight and weaning weight of each piglet were recorded as well as the daily feed intake per litter.

### 2.3. Behavioral Observations

Twelve supervision cameras (Model: DS-2CD3T86FWDV2-I3S, Hikvision Digital Technology Co., Ltd., Hangzhou China) were placed 2.5 m high above ground level to record sow and piglet behavior continuously during the whole experimental period. Each camera recorded the behavior of two sows and their piglets. The ethogram of observed behaviors was partially based on Van Beirendonck et al. [4] and Chidgey et al. [18] (Table 1). The day of farrowing was defined as day 0. The behavior of sows on 6 (day −6) and 3 (day −3) days before parturition and days 3, 7, 14 and 21 after parturition was scan sampled every 5 min from 08:00 to 11:00 and from 14:00 to 17:00. In addition, the behavior of sows on the day of parturition (12 h before and after the birth of the first piglet) was focally observed. Piglets from each sow were behaviorally scan sampled in a group level on days 3, 7, 14 and 21 after parturition. On each day, the scan sampling interval and observational periods were the same as the behavioral observation of the sows. 

### 2.4. Statistical Analysis

Data were analyzed using the statistical software JMP 14.1 (SAS Institute Inc., Cary, NC, USA). All the data of percentages such as total mortality, crushing rate, and behavioral data collected by scan sampling and calculated from focal sampling were arcsine square rooted to meet the assumption of normalization. One-way Analysis of Variance (ANOVA) was used to test the farrowing system effect on the average birth weight, weaning weight and daily feed intake of piglets. The farrowing system effect on the piglet production and loss data such as the number of piglet total born, born alive, stillbirth, weaned, and crushed were examined with non-parametric Kruskal-Wallis tests. As there was the companion of piglets after parturition, the behavior of sows before and after parturition was analyzed separately. For the behavior of sows before and after parturition and the behavior of piglets after parturition, the repeated-measure ANOVA was used to examine the effects of the farrowing system, day, and their interaction. The observational days was taken as repeated measures. The duration of each behavior of sows during the 12 h before and after parturition were recorded, calculated, and expressed as percentages. One-way ANOVA was performed to test the farrowing system effects on the crushing rate and each kind of behavior (separately for before and after parturition). *p* < 0.05 is taken as significantly different. A tendency of difference was defined when 0.05 ≤ *p* < 0.10 was observed.

## 3. Results

### 3.1. Piglets Production and Loss

The piglets from three farrowing systems had similar birth weight (*p* = 0.83) and daily feed intake (*p* = 0.77). The weaning weight of piglets from FFS group tended to be higher than the piglets from the other two farrowing systems (*p* = 0.08). No statistical difference was found in the number of piglet total born (*p* = 0.24), born alive (*p* = 0.40), and stillborn (*p* = 0.67) between the treatments (Table 2). The number of piglets crushed in the FFS and FFSN group was respectively 0.6 ± 0.7 (mean ± SD) and 0.9 ± 1.1 (mean ± SD) which were numerically higher comparing with the FC group (mean ± SD, 0.1 ± 0.3) but without a statistical difference (*p* = 0.40, Table 2). Similar result was seen for the piglet crushing rate (%, mean ± SD, FC 1.0 ± 3.0, FFS 5.5 ± 6.6, FFSN 9.6 ± 13.4, *p* = 0.25, Table 2). The weaning number of piglets seemed lower in the FFSN group which was 7.7 ± 1.8 (mean ± SD) compared with the FC (mean ± SD, 9.6 ± 1.9) and FFS (mean ± SD, 9.4 ± 1.7) group but still without a statistical difference (*p* = 0.12, Table 2). The FC group (%, mean ± SD, 7.5 ± 8.6) tended to have a lower piglet total mortality rate compared with the other two groups (%, mean ± SD, FFS 17.1 ± 12.3, FFSN 19.6 ± 11.8) (*p* = 0.07, Table 2). The detailed information of piglet production and loss was provided in Table S1.

### 3.2. Sow Behavior Before Farrowing

On both 6 and 3 days before parturition, FFS sows spent significantly more time lying compared with the sows from the other two treatments (*p* < 0.01), whereas FC sows performed the most sitting behavior among the three treatments (*p* < 0.01) (Table 3). In terms of the behavior of standing (*p* < 0.01) and exploring (*p* < 0.01), the three treatments were significantly different from each other as the FFSN sows stood and explored the most and followed by the FFS and FC sows (Table 3). The FC sows conducted significantly more abnormal behaviors such as sham chewing (*p* < 0.01) and biting fixtures (*p* < 0.01) compared to the sows from the other two treatments (Table 3). No day effect was found in all the behaviors observed before farrowing (*p* > 0.05, Table 3). There was no interaction between treatment and day in all behaviors (*p* > 0.05, Table 3). When it was close to the time of farrowing (within 12 h before farrowing), the FC sows performed the most lying (*p* = 0.04) and the least standing (*p* < 0.01) and explorative (*p* < 0.01) behavior compared to the sows from the other two treatments (Table 4). The FC sows performed more sitting (*p* < 0.01) and biting fixture (*p* < 0.01) behavior than the FFS and FFSN sows (Table 4).

### 3.3. Sow Behavior after Farrowing

During the three weeks after parturition, there were significant effects of treatment and day in all behaviors observed (*p* < 0.05, Table 5). The effects of interaction between treatment and day were observed for the sitting and standing behavior (*p* < 0.01, Table 5). Specifically, the FC sows spend more time lying (days 7 and 14) and sitting (days 14 and 21) while less time standing (days 7, 14, and 21) compared with the FFS and FFSN sows (*p* < 0.01) except that there was no difference in the lying behavior between the FC and FFSN sows on day 14 (*p* > 0.05) (Table 5). The three treatments were significantly different from each other in explorative behavior with the FFSN sows exploring the most followed by the FFS and FC sows on all observational days, except that the FFS and FFSN sows had a similar time of exploring on day 3 (*p* < 0.01, Table 5). In addition, on day 3, the FFSN sows exhibited less sham chewing compared with the sows from the other two treatments (*p* < 0.05), whereas no treatment effect was found in the biting fixture behavior (*p* > 0.05) (Table 5). On days 7, 14, and 21, the FC sows were found to perform the most sham chewing and biting fixture behavior compared with the sows from the other two treatments (*p* < 0.05), except that no difference was observed in the biting fixture behavior between the FC and the FFS sows on day 21 (*p* > 0.05) (Table 5). In terms of the behavioral results collected by focal observation during the 12 h after parturition, no difference was seen between treatments in the lying and sitting behavior (*p* > 0.05) and the FC sows conducted less standing behavior than the sows from the other two treatments (*p* < 0.01) (Table 4).

### 3.4. Piglet Behavior

Significant treatment (*p* < 0.05) and day (*p* < 0.01) effects were exhibited in all piglet behaviors except that no treatment effect was seen in the suckling behavior (*p* > 0.05) (Table 6). Significant interactions between treatment and day were observed in the lying (*p* = 0.03), locomotion (*p* < 0.01) and aggressive (*p* < 0.01) behavior (Table 6). In detail, the piglets in the FFSN group spend less time lying compared with the piglets in the FC and FFS group on day 14 and the lying behavior was performed more by the piglets in the FC group compared with the piglets in the other two groups on day 21 (*p* < 0.05, Table 6). The difference between treatments in standing behavior on day 21 was the same as lying behavior on the same day (*p* < 0.05, Table 6). In terms of locomotion behavior, on day 7, the FFSN piglets conducted more than the FC piglets (*p* < 0.05) and piglets from these two treatments had no difference compared with the FFS piglets (*p* > 0.05); on day 14, the FFSN piglets performed more than the piglets from the other two treatments (*p* < 0.05); on day 21, the FC piglets had less locomotion time than the FFS and FFSN piglets (*p* < 0.05) (Table 6). The FFSN piglets had significantly more explorative behavior than the FC and FFS piglets on days 14 and 21 (*p* < 0.05, Table 6). The FC piglets had the most whereas the FFSN piglets had the least aggressive behavior among the three treatments on day 14 (*p* < 0.05, Table 6). The FC piglets were less likely to be in close proximity to the sows than the FFSN piglets on day 14 while, on day 21, both the FFS and FFSN piglets had a higher chance to be closer with the sows (*p* < 0.05, Table 6). No difference was seen in terms of the behaviors on the days that were not mentioned in the above description (*p* > 0.05, Table 6).

## 4. Discussion

As we can see from the results, the piglet loss tended to be increased by removing crate confinement. Using free farrowing pens could increase the activity, enrich behavioral pattern, and decrease abnormal behavior of sows as well as elevate the activity level of piglets. In addition, providing nest materials was found to promote the explorative behavior of both sows and piglets.

In terms of piglet loss under free farrowing systems, a big variability in total mortality has been reported ranging from 9% [19] to 30% [12] under experimental conditions. In the present study, the piglet total mortality of 17.1% and 19.6% respectively for the FFS and FFSN group is within the mortality range of previous studies. The numerically higher piglet total mortality in the free farrowing pens compared with the farrowing crate pens indicating a potential big financial loss in the industry [20]. However, Singh et al. [21] found no difference in piglet mortality between traditional farrowing crate pens and free farrowing pens from day 3 of lactation until weaning based on the recording of 672 litters in total. With the banning of the farrowing crate in several countries, more information was collected in practice with a promising result of similar total piglet mortality between non-confined and crate farrowing systems (in Switzerland, loose = 17.2% from 18824 litters and crates = 18.1% from 44837 litters) [2,22]. Based on the aforementioned data and the concerning of sow welfare, the free farrowing system should be seriously considered. However, concerning the potential financial loss according to the piglet loss result in the current study, improvements of the farrowing systems and fine managements should still be intensively investigated. 

It has been proved that commercial pigs still retain the nest-building behavior before parturition [23,24]. Locomotion is an important aspect of nesting behavior [25]. Before parturition, sows in the free farrowing pens conducted more standing and explorative behavior compared with the sows restricted in the farrowing crates and this observation is in line with the study of Jarvis et al. [6]. The explorative behavior of sows was enhanced when nest materials were provided. Cronin et al. [26] observed a similar effect of the straw provision in farrowing pens during the 24 h pre-partum. Studnitz et al. [27] reported that when given the opportunity, pigs are strongly motivated to explore their environment. The increased space allowance and/or nest material provision allowed the sows to perform their instinct behavior and thus reduced the conduction of stereotypic behaviors such as sham chewing and biting fixture which were commonly observed in sows kept in crates [7].

On day 3 post-partum, the lack of difference in the lying, sitting and standing behavior of sows between treatments was in line with the study of Chidgey et al. [18] who observed no difference in those behaviors between sows housed in crate and loose farrowing systems during days 1-6 after parturition. Similar amounts of time lying of sows in the confined or loose environment during the first 3 days after farrowing was also observed by Hales at el. [28]. During the 12h after farrowing, the lying and sitting behavior of sows were similar across different farrowing systems with more than 90% of the time spent lying. Jensen [29] reported that, under semi-natural conditions, sows spent 90% of time lying during the first 2 days post-farrowing which is in agreement with our study. Therefore, there is a period of at least three days of low activity of sows after parturition. In terms of stereotypic behavior, occupying more than 10% of time indicates welfare impairments [30]. In the present study, the total time spent performing stereotypies (biting fixture and sham chewing) on day 3 post-partum was 7.8%, 6.2%, and 4.6% respectively for the FC, FFS, and FFSN sows. Therefore, due to the significant proportion of lying behavior and relatively low occurrence of stereotypies on day 3 post-partum, few days farrowing crate confinement right after farrowing might have limited welfare concern for sows while assuring fewer piglets being crushed [18,31]. Starting from day 7 onwards, sows in free farrowing pens showed less lying and more standing behavior. Valros et al. [32] found the sows in the loose-housed environment had an increase in activity after the first-week post-farrowing. These results indicated that increasing space allowance for postpartum sows might be necessary at least 7 days after farrowing. The explorative behavior of sows was more pronounced by providing bedding material after 7 days post-farrowing indicating the necessity of bedding material provision.

In terms of the behavior of piglets, differences were mainly observed starting from day 14 post-farrowing except that the FFSN piglets conducted more locomotion behavior than the FC piglets starting from day 7 post-farrowing. Similar results have been reported that outdoor piglets conducted more locomotive and explorative behavior than piglets from indoor farrowing crates [33,34]. Van Beirendonck et al. [4] have indicated that there was an association between the postures and activities of sows and the behavior of their piglets in farrowing crate systems. They specifically noticed that piglets prefer resting when sows are resting, whereas when sows were standing up, piglets performed more running around behavior. It is noteworthy that the differences in piglet behavior across treatments started to show when the activity of sows elevated on day 7 post-farrowing onwards. Therefore, the increased locomotion behavior of piglets in the FFS/FFSN group seemed likely due to the elevated activity of their corresponding sows. As it is quite difficult to distinguish aggressive behavior from playing behavior, our result of aggressive behavior might partially include some active playing of piglets. Hence, we will not draw much conclusion here in terms of the aggressive behavior. The enriched behavioral pattern of sows in free farrowing systems and the environmental enrichment factor of nest material might direct the piglets towards more positive behavior such as locomotion and exploration.

## 5. Conclusions

Farrowing systems without crates has beneficial influences on the behavior of sows and piglets but with potential higher piglet loss. Adding nest material in the farrowing pens is suggested as an environmental enrichment for sows and piglets. Sow welfare, piglet survival, and fine managements should all be considered in order to successfully introduce the free farrowing system into the industry.

## Figures and Tables

**Table 1:** Ethogram of behavior recorded during observations of sows and piglets.

Animal	Behavioral Parameter	Description
Common	Lying	Lying laterally (Lying flat on one side with a shoulder on the floor and udder exposed) and lying ventrally (Lying on sternum/belly, the udder is partially or totally obscured)
	Standing	Maintaining an upright body position with 4 legs supporting sow body weight
Sow behavior	Explorative behaviors	Nosing floor including slatted floor, solid floor, and soft bed
	Sham chewing	Chewing air, chewing with no food in the mouth
	Biting fixture	Biting at any of the fixtures within the crate or pen (e.g., bars, trough)
	Sitting	Partly erect on front legs with the hindquarters in contact with the floor
Piglet behavior	Locomotion	Walking, trotting on the floor
	Suckling	Piglet is suckling from a teat
	Explorative behaviors	Nosing floor including slatted floor, solid floor, and soft bed
	Aggressive behavior	Forceful fighting, pushing with the head or biting littermates in a violent manner
	Proximity to sow	In the range of 20 cm from the sow

**Table 2:** Piglet production and loss for sows in different farrowing systems.

Parameter	FC	FFS	FFSN	SD	*p*-Value
Average birth weight (kg)	1.5	1.6	1.5	0.2	0.83
Average weaning weight (kg)	6.3	6.8	6.3	0.5	0.08
Average daily feed intake (g/d)	6.5	6.5	6.8	0.6	0.77
Total born	10.5	11.4	9.6	2.2	0.24
Born alive	9.8	10.3	8.7	2.1	0.40
Stillbirth	0.8	1.1	0.9	0.9	0.67
Weaning number	9.6	9.4	7.7	2.1	0.12
Total mortality (%)	7.5	17.1	19.6	12.5	0.07
Crushing number	0.1	0.6	0.9	0.8	0.40
Crushing rate (%)	1.0	5.5	9.6	9.5	0.25

FC—farrowing crate system; FFS—free farrowing pen with sloping walls; FFSN—free farrowing pen with sloping walls and nest materials; SD—standard deviation.

**Table 3:** Sow behavior on the 6 and 3 d before parturition in different farrowing systems.

Behavior (%)	Treatment	Day		SD	*p*-Values		
		−6	−3		Treatment	Day	Treatment × Day
Lying	FC	80.7 ^b^	81.6 ^b^	4.5	<0.01	0.50	0.33
	FFS	86.3 ^a^	86.3 ^a^				
	FFSN	81.9 ^b^	78.6 ^b^				
Sitting	FC	14.4 ^a^	14.4 ^a^	5.7	<0.01	0.48	0.62
	FFS	4.2 ^b^	4.2 ^b^				
	FFSN	2.6 ^b^	3.4 ^b^				
Standing	FC	4.9 ^c^	4.0 ^c^	5.7	<0.01	0.97	0.61
	FFS	9.7 ^b^	9.5 ^b^				
	FFSN	15.5 ^a^	17.1 ^a^				
Explorative behavior	FC	1.9 ^c^	2.6 ^c^	6.7	<0.01	0.51	0.57
	FFS	7.7 ^b^	7.3 ^b^				
	FFSN	15.9 ^a^	16.5 ^a^				
Sham chewing	FC	8.9 ^a^	9.2 ^a^	3.7	<0.01	0.67	0.92
	FFS	3.4 ^b^	3.6 ^b^				
	FFSN	3.0 ^b^	3.0 ^b^				
Biting fixture	FC	5.6 ^a^	6.9 ^a^	2.6	< 0.01	0.77	0.17
	FFS	2.4 ^b^	1.8 ^b^				
	FFSN	2.6 ^b^	3.0 ^b^				

FC—farrowing crate system, N = 8; FFS—free farrowing pen with sloping walls, N = 7; FFSN—free farrowing pen with sloping walls and nest materials, N= 7; SD—standard deviation. ^a, b, c^—Values with different superscripts differ significantly (*p* < 0.05) among different treatments (columns) within the same day.

**Table 4:** Sow behavior within 12 h before and after farrowing in different farrowing systems.

Time	Behavior (%)	Treatment			SD	*p*-Values
		FC	FFS	FFSN		
Before farrowing	Lying	76.1 ^a^	69.1 ^b^	68.2 ^b^	7.0	0.04
	Sitting	7.0 ^a^	4.7 ^b^	4.2 ^b^	2.1	<0.01
	Standing	17.0 ^b^	26.2 ^a^	27.6 ^a^	7.7	<0.01
	Explorative behavior	12.1 ^b^	16.3 ^a^	19.1 ^a^	4.5	<0.01
	Biting fixture	6.2 ^a^	3.9 ^b^	3.5 ^b^	1.6	<0.01
After farrowing	Lying	95.0	92.7	92.3	2.5	0.10
	Sitting	2.7	2.2	2.2	0.8	0.33
	Standing	2.2 ^b^	5.4 ^a^	5.6 ^a^	2.4	<0.01

FC—farrowing crate system, N = 8; FFS—free farrowing pen with sloping walls, N = 7; FFSN—free farrowing pen with sloping walls and nest materials, N = 7; SD—standard deviation. ^a, b^—Values with different superscripts differ significantly (*p* < 0.05) among different treatments (rows).

**Table 5:** Sow behavior during the first three weeks after farrowing in different farrowing systems.

Behavior (%)	Treatment	Day				SD	*p*-Values		
		3	7	14	21		Treatment	Day	Treatment × Day
Lying	FC	85.9	83.5 ^a^	72.7 ^a^	71.5	8.6	<0.01	<0.01	0.10
	FFS	86.1	72.0 ^b^	65.5 ^b^	72.6				
	FFSN	85.7	75.2 ^b^	66.5 ^ab^	69.4				
Sitting	FC	4.9	9.7	21.4 ^a^	18.4 ^a^	6.0	<0.01	<0.01	<0.01
	FFS	5.0	10.3	9.7 ^b^	7.3 ^b^				
	FFSN	3.6	9.5	7.7 ^b^	7.5 ^b^				
Standing	FC	8.5	6.8 ^b^	5.9 ^b^	9.9 ^b^	7.9	<0.01	<0.01	<0.01
	FFS	8.9	17.7 ^a^	24.8 ^a^	20.0 ^a^				
	FFSN	10.7	15.3 ^a^	26.0 ^a^	23.0 ^a^				
Explorative behaviors	FC	2.1 ^b^	3.3 ^c^	3.3 ^c^	4.0 ^c^	5.6	<0.01	<0.01	0.07
	FFS	3.6 ^a^	8.5 ^b^	9.9 ^b^	9.9 ^b^				
	FFSN	6.3 ^a^	11.7 ^a^	18.5 ^a^	15.9 ^a^				
Sham chewing	FC	4.0 ^a^	7.5 ^a^	8.7 ^a^	9.2 ^a^	3.0	<0.01	0.04	0.08
	FFS	3.8 ^a^	3.4 ^b^	4.0 ^b^	3.2 ^b^				
	FFSN	1.8 ^b^	2.4 ^b^	2.8 ^b^	2.6 ^b^				
Biting fixture	FC	3.8	5.4 ^a^	5.9 ^a^	6.6 ^a^	2.4	<0.01	0.01	0.74
	FFS	2.4	2.8 ^b^	3.8 ^b^	5.6 ^ab^				
	FFSN	2.8	2.4 ^b^	3.2 ^b^	3.6 ^b^				

FC—farrowing crate system, N = 8; FFS—free farrowing pen with sloping walls, N = 7; FFSN—free farrowing pen with sloping walls and nest materials, N= 7; SD—standard deviation. ^a, b, c^—Values with different superscripts differ significantly (*p* < 0.05) among different treatments (columns) within the same day.

**Table 6:** Piglet behavior during the first three weeks after farrowing in different farrowing systems.

Behavior (%)	Treatment	Day				SD	*p*-Values		
		3	7	14	21		Treatment	Day	Treatment × Day
Lying	FC	72.7	70.9	66.1 ^a^	61.1 ^a^	7.8	<0.01	<0.01	0.03
	FFS	74.5	69.8	64.4 ^a^	57.2 ^b^				
	FFSN	73.4	63.1	59.6 ^b^	56.5 ^b^				
Standing	FC	8.9	7.9	6.9	7.8 ^a^	1.6	<0.01	<0.01	0.12
	FFS	8.4	7.4	6.2	5.6 ^b^				
	FFSN	9.9	7.7	6.9	6.1 ^b^				
Locomotion	FC	18.1	21.4 ^b^	27.4 ^b^	31.7 ^b^	7.4	<0.01	<0.01	< 0.01
	FFS	17.7	22.7 ^ab^	29.1 ^b^	36.9 ^a^				
	FFSN	17.1	23.4 ^a^	33.1 ^a^	37.9 ^a^				
Explorative behaviors	FC	9.7	11.2	11.7 ^b^	14.0 ^b^	3.2	<0.01	<0.01	0.06
	FFS	10.9	12.3	11.4 ^b^	15.1 ^b^				
	FFSN	11.6	12.6	15.8 ^a^	19.8 ^a^				
Suckling	FC	20.3	18.1 ^ab^	16.8	16.2	2.1	0.63	<0.01	0.13
	FFS	19.7	17.5 ^a^	16.0	16.8				
	FFSN	20.0	18.6 ^b^	17.8	18.3				
Aggressive behavior	FC	2.9	4.3	6.4 ^a^	2.6	1.5	0.04	<0.01	<0.01
	FFS	3.1	3.9	4.6 ^b^	2.3				
	FFSN	3.4	3.7	2.8 ^c^	2.4				
Proximity to sow	FC	79.8	73.3	69.3 ^b^	61.5 ^b^	6.4	0.02	<0.01	0.11
	FFS	79.5	74.3	72.8 ^ab^	66.5 ^a^				
	FFSN	79.6	73.2	74.7 ^a^	67.3 ^a^				

FC—farrowing crate system, N = 8; FFS—free farrowing pen with sloping walls, N = 7; FFSN—free farrowing pen with sloping walls and nest materials, N = 7; SD—standard deviation. ^a, b, c^—Values with different superscripts differ significantly (*p* < 0.05) among different treatments (columns) within the same day.

## Supplementary Materials

The following are available online at https://www.mdpi.com/2076-2615/10/2/320/s1, Table S1: The piglet production and loss of sows in different farrowing systems (farrowing crate, FC; free farrowing pen with sloping walls, FFS; free farrowing pen with sloping walls and nest materials, FFSN).
