# Supplementary material for: Effects of Different Farrowing Environments on the Behavior of Sows and Piglets
Source: Animals (Basel). 2020 Feb 18;10(2):320. doi: 10.3390/ani10020320 (PMC7070988; doi:10.3390/ani10020320)
Supplement: Supplementary file 1 [file animals-10-00320-s001.pdf]

Supplementary File: Effects of Different Farrowing Environments on the Behavior of Sows and Piglets

Xiaojun Zhang †, Congcong Li †, Yue Hao and Xianhong Gu *

Table 1. The piglet production and loss of sows in different farrowing systems.

Groups	Total Born	Born Alive	Still Births	Weaning Numbers	Crushing Numbers	Total Deaths
FC1	10	10	0	10	0	0
FC2	10	9	1	9	0	1
FC3	9	9	0	9	0	0
FC4	16	13	3	12	1	4
FC5	12	12	0	12	0	0
FC6	11	11	0	11	0	0
FC7	7	6	1	6	0	1
FC8	9	8	1	8	0	1
FFS1	10	9	1	9	0	1
FFS2	12	12	0	12	0	0
FFS3	15	13	2	10	2	5
FFS4	11	11	0	10	1	1
FFS5	12	9	3	9	0	3
FFS6	9	7	2	6	1	3
FFS7	11	11	0	10	0	1
FFSN1	6	5	1	5	0	1
FFSN2	11	10	1	10	0	1
FFSN3	11	10	1	8	2	3
FFSN4	9	8	1	5	3	4
FFSN5	10	10	0	8	1	2
FFSN6	10	9	1	9	0	1
FFSN7	10	9	1	9	0	1

FC—farrowing crate; FFS—free farrowing pen with sloping walls; FFSN—free farrowing pen with sloping walls and nest materials.
